# The Biased Nucleotide Composition of HIV-1 Triggers Type I Interferon Response and Correlates with Subtype D Increased Pathogenicity

**DOI:** 10.1371/journal.pone.0033502

**Published:** 2012-04-18

**Authors:** Nicolas Vabret, Marc Bailly-Bechet, Valérie Najburg, Michaela Müller-Trutwin, Bernard Verrier, Frédéric Tangy

**Affiliations:** 1 Unité de Génomique Virale et Vaccination, CNRS URA-3015, Institut Pasteur, Paris, France; 2 Laboratoire de Biométrie et Biologie Evolutive, CNRS UMR 5558, Université Claude Bernard Lyon 1, Villeurbanne, France; 3 Unité de Régulation des Infections Rétrovirales, Institut Pasteur, Paris, France; 4 Institut de Biologie et Chimie des Protéines, CNRS FRE 3310, University of Lyon 1, Lyon, France; Inserm, France

## Abstract

The genome of human immunodeficiency virus (HIV) has an average nucleotide composition strongly biased as compared to the human genome. The consequence of such nucleotide composition on HIV pathogenicity has not been investigated yet. To address this question, we analyzed the role of nucleotide bias of HIV-derived nucleic acids in stimulating type-I interferon response in vitro. We found that the biased nucleotide composition of HIV is detected in human cells as compared to humanized sequences, and triggers a strong innate immune response, suggesting the existence of cellular immune mechanisms able to discriminate RNA sequences according to their nucleotide composition or to detect specific secondary structures or linear motifs within biased RNA sequences. We then extended our analysis to the entire genome scale by testing more than 1300 HIV-1 complete genomes to look for an association between nucleotide composition of HIV-1 group M subtypes and their pathogenicity. We found that subtype D, which has an increased pathogenicity compared to the other subtypes, has the most divergent nucleotide composition relative to the human genome. These data support the hypothesis that the biased nucleotide composition of HIV-1 may be related to its pathogenicity.

## Introduction

Without treatment, most individuals infected with HIV will develop AIDS as a consequence of years of chronic and excessive activation of their immune system [1]. The generalized immune activation in AIDS consists in over expression of activation markers, excessive proliferation and apoptosis of CD4 and CD8 T cells, NK cells, B cells and macrophages, in addition to a strong type I interferon response, which is maintained throughout the chronic phase of infection [2]. Because the virus replicates preferentially in activated CD4 T cells, chronic activation supports viral replication by providing targets. A number of mechanisms have been suggested to contribute to immune activation in AIDS pathogenesis [3], [4], such as i) the innate and adaptive response to HIV and its antigens, ii) the direct effect of viral proteins on cell receptors like Env-CD4/CCR5 or TCR down-regulation by Nef, iii) the activation of TLRs by microbial products leaking from the damaged gut mucosa, iv) the frequency of opportunistic infections, v) increased levels of proinflammatory and pro-apoptotic cytokines, vi) depletion of CD4 T regulatory cells.

Viral nucleic acids trigger an antiviral innate immune response in infected cells, which contributes to activating the immune system [4], [5], [6]. Cytoplasmic accumulation of HIV nucleic acids has been shown to be responsible for the majority of T-cell apoptosis during HIV infection [7]. IFN-α/β is the principal mediator of antiviral innate immunity. Different forms of viral RNA molecules can trigger IFN-α/β stimulation, either single or double stranded, naked or combined with capsid proteins [8], [9], [10], [11]. Several cytosolic receptors have been identified that recognize HIV-1 patterns [12], [13]. Plasmacytoid dendritic cells (pDCs) produce large amounts of IFN-α/β in response to microbial stimuli. During the acute phase of HIV infection, pDCs are the main producers of IFN-α/β. They recognize viral ssRNA via TLR7, and dsDNA via TLR9 [14], [15]. This antiviral response contributes to containing the acute infection. In primate models of lentiviral infection, pDCs produce large amounts of IFN-α/β during the acute phase of infection, whatever a pathogenic or non-pathogenic outcome [4], [6]. However, during the chronic phase of infection, viral RNA, which is still produced in infected cells, stimulates IFN-α/β production on the long term. A recent study reported that in chronically HIV-infected patients IFN-α colocalized only with few pDCs, but rather with other TLR7 negative cells [16].

The genomes of HIV and most lentiviruses present a particularly biased nucleotide composition compared to that of their primate hosts, with as much as 35% adenosine in HIV-1 RNA genome. As a consequence, the average amino acid composition is different and the synonymous codon usage is also modified [17], [18]. Lentiviral biased nucleotide composition has been explained by dNTP pool imbalance during reverse transcription [19], [20] and by the antiviral activity of the cellular Apobec 3G (A3G) cytidine deaminase, which mutates G to A in HIV provirus [21] thus impacting HIV-1 evolution and drug resistance [22], [23], although the counteracting activity of the viral Vif protein reduces Apobec impact on the nucleotide bias of HIV genome [20].

Such biased nucleotide composition obviously has direct consequences on genome structure and stability, but its effects on viral biology have not been investigated yet. We wondered whether the strongly divergent nucleotide composition of HIV-1 nucleic acids, as compared to host, plays a role in triggering antiviral innate response. To address this question, we compared the capacity of HIV-1 RNA molecules corresponding to the three major viral genes, either wild type or artificially unbiased, to modulate IFN-α/β synthesis in a sensitive in vitro assay in human cells. We also tested a set of small RNA fragments covering the entire HIV-1 genome for their capacity to activate IFN-α/β response according to their nucleotide composition. We then performed a large-scale bioinformatics study to compare the divergence in nucleotide composition between all subtypes of HIV-1 group M viruses. Altogether, this work shows that AU-rich biased HIV-1 sequences, mainly located within *gag, pol, env* genes, strongly stimulate IFN-α/β response, and that HIV-1 subtype D, which has an increased pathogenicity, has a more biased sequence.

## Results

### The Biased Nucleotide Composition of HIV-1 RNA Stimulates IFN-α/β Synthesis

**Figure 1:**
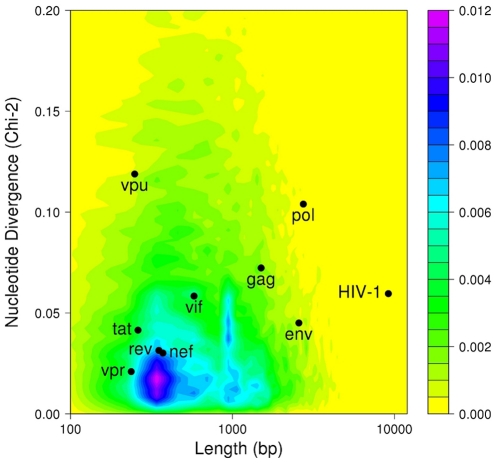
Most HIV-1 genes are divergent from human coding sequences. The distribution of human coding sequences is represented according to their length (log scale) and nucleotidic divergence, as measured by the Chi-square distance relative to average composition of all human coding sequences. The colored background shows the density of human coding sequences as indicated on the z-scale. Black dots represent either single HIV-1 genes or the entire HIV-1 genome. Note the distinguishable positions of the *gag*, *pol*, *env* and *vpu* genes.

**Figure 2:**
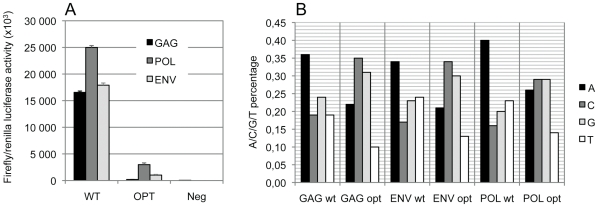
Humanization of HIV-1 genes strongly reduces their ability to induce IFN-α/β. (A) Type-I interferon stimulation was measured as the ISRE-dependant expression of luciferase activity in HEK 293 T cells upon transient transfection of HIV-1-derived RNA molecules. The ISRE reporter activity is the ratio of firefly luciferase activity to control renilla luciferase activity in triplicate experiments. In vitro transcribed RNA molecules corresponding to the wild type sequence of the three HIV-1 major genes induced a strong response while their humanized version induced a tenfold lower response. (B) Nucleotide composition of HIV-1 RNA fragments expressed as the A/C/G/U frequencies.

To first analyze the divergence between HIV and human nucleotide composition, we plotted the distribution of human coding sequences according to their length and nucleotide divergence, as measured by the Chi-square distance relative to average nucleotide A/C/G/T frequency of all human coding sequences (Figure 1). This analysis shows that the majority of human coding sequences are gathered in a group of relatively small size sequences with homogenous nucleotide composition. Comparing in the same analysis HIV-1 nucleotide composition to the human genome highlights that *gag, pol, env* and *vpu* genes are highly divergent from human sequences (black dots in Figure 1). To investigate the role of such biased nucleotide composition of HIV genome on immune activation, we analyzed the ability of HIV-1-derived RNA fragments, whose nucleotide sequence was either wild type (WT) or artificially optimized to human codon usage, to induce IFN-α/β synthesis *in vitro* in epithelial-like HEK 293 T cells (ATCC, CRL-11268). We chose these cells because they are easily transfectable and produce IFN-α/β in response to HIV-1 infected cells although they lack TLR7 [13]. The three major HIV-1 genes *gag, pol, env* (*gag, pol* from *hxb2* sequence and *env* from a primary isolate sequence) and their corresponding versions optimized towards human codon bias (Gag opt, Pol opt, Env opt) were *in vitro* transcribed with T7 RNA polymerase into uncapped and unpolyadenylated RNA fragments. These RNA fragments were co-transfected into HEK 293 T cells together with a reporter plasmid containing the luciferase gene fused downstream of five interferon-stimulated response elements (ISRE-luciferase). A low amount of a plasmid harboring a thymidine kinase (Tk) promoter just upstream of the renilla luciferase gene was also co-transfected to allow normalization between wells. In a first set of experiments, we performed a dose-response analysis for each RNA molecule tested to determine the highest non-toxic dose that did not affect the level of renilla expression. A unique dose of 20 ng that was not toxic and did not affect, or only moderately, transfection efficiency was chosen for transfecting all RNA fragments (Figure S1). The reporter activity, reflecting the induction of IFN-α/β response, was calculated as the ratio of firefly luciferase activity to reference renilla luciferase activity measured in cell lysates 20 hours after transfection (Figure 2A). While the WT versions of Gag, Pol or Env RNAs induced a strong IFN-α/β expression, this capacity was abolished in their humanized versions, which induced very low levels of IFN-α/β. Although both type of sequences shared the same coding information, transfecting uncapped and unpolyadenylated RNA molecules in this experiment allowed evaluating the role of RNA molecules in the absence of expressed proteins. Moreover, to ensure that no protein is expressed from RNA, we deleted the first nucleotide of the AUG codon in each construct. The nucleotide composition of wild type and humanized HIV-1 RNA fragments used in this experiment reveals that humanization results essentially in reducing A/U and increasing G/C contents (Figure 2B). Our observation demonstrates that the biased nucleotide composition of HIV synthetic RNA molecules, or its structural consequence, is detected in human cells and triggers a strong innate immune response.

### A-rich Regions of HIV-1 Genome are Responsible for IFN-α/β Stimulation

**Figure 3:**
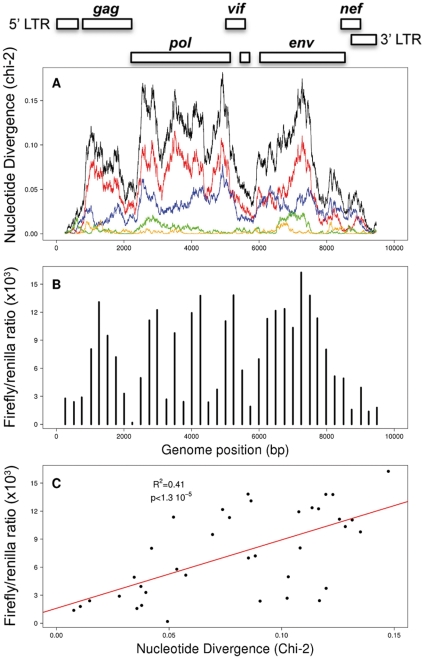
The nucleotidic divergence of HIV-1 RNA fragments correlates with their ability to stimulate IFN-α/β. **A**) The black line shows the nucleotide divergence of HIV-1 compared to human genome, as measured by a Chi-square distance in a 500 bp sliding window along the HIV-1 *hxb2* genome. The individual contributions of A, C, G, and U nucleotides to this divergence are shown respectively in red, blue, yellow and green. The HIV-1 genome map is schematically represented above the figure. **B**) Histogram represents the ISRE reporter activity as the ratio of firefly luciferase activity to control renilla luciferase activity determined in HEK 293 T cells cotransfected with HIV-1 RNA fragments and pISRE-Luc reporter. **C**) Correlation between the nucleotide divergence (x-axis) and the luciferase activation (y-axis) of the 38 RNA fragments from HIV-1 hxb2 genome. Each point corresponds to the average of four replicates and the correlation coefficient was computed only on these averages to not artefactually lower the p-value.

To investigate whether shorter specific regions within HIV-1 genome are responsible for IFN-α/β stimulation, we first looked for hyper-biased region on the whole genome. For that, we computed the Chi-square distance between the A/C/G/U frequencies of a sliding window 500 nt wide along the genome of HIV-1 clone *hxb2* and the corresponding frequencies of the entire coding sequences of the human genome (Figure 3A). As expected from Figure 1, we found the most divergent regions located within the three large viral genes (*gag, pol, env*), while overlapping coding regions and cis-active regulatory sequences are unbiased. Since adenosine enrichment contributes to most of the divergence (red line in Figure 3A), these “hot spots” of divergence are likely the footprint of adenosine accumulation during low-fidelity reverse transcription and by the action of cytidine deaminases. This highlights that the long open reading frames (ORF) of lentiviruses (gag, pol, env) can accumulate more synonymous mutations than the rest of the genome without impairing viral fitness. They reflect the tendency of lentiviral genomes to become A-rich through evolution [24].

**Figure 4:**
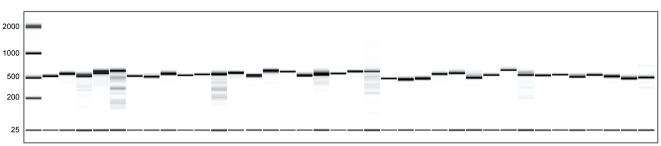
. **Overlapping RNA fragments covering the entire genome of HIV-1** ***hxb2*** **.** PCR fragments of approximately 500 bp, with overlaps of 250 bp, and covering the entire genome of HIV-1 *hxb2* were *in vitro* transcribed with T7 RNA polymerase into a set of uncapped and unpolyadenylated RNA fragments. After purification RNA fragments were migrated on Agilent-2100 Small RNA chips. A ladder on the left indicates the size in nucleotides.

To determine then the local distribution of immune activating sequences along the viral genome, we measured the ability of a set of RNA fragments covering the entire genome of HIV-1 clone *hxb2* to induce IFN-α/β synthesis *in vitro* in the same system as used above. We generated 38 PCR fragments of approximately 500 bp, with overlaps of 250 bp, and covering the entire genome of HIV-1 *hxb2* (primers used are listed in Table S1). These DNA fragments were *in vitro* transcribed with T7 RNA polymerase into a set of overlapping uncapped and unpolyadenylated RNA fragments. The quality and integrity of RNA fragments was controlled by migration on electrophoresis chips (Figure 4). These RNA fragments were co-transfected into HEK 293 T cells together with the reporter ISRE-luciferase plasmid, and luciferase activity was measured 20 h after transfection. Again, we performed a dose-response analysis for each RNA molecule to determine the highest non-toxic dose that did not affect the level of renilla expression. A unique dose of 12 ng that was not toxic and did not affect transfection efficiency was chosen for transfecting all RNA fragments (Figure S1). The ability of each fragment to induce an interferon response, calculated as the ratio of firefly luciferase activity to reference renilla luciferase activity (Figure 3B), was compared to its divergence in nucleotide composition with the human genome composition (Figure 3A, black line). The comparison highlights that RNA fragments that have the greatest divergence in nucleotide composition (mainly due to adenosine enrichment) are the most potent stimulators of IFN-α/β response (Figure 3C, p  =  1.3 10^−5^, Pearson correlation test, R^2^  =  0,41). Although the p-value of this correlation is very strong, some fragments do not follow the global trend, particularly within the *pol* gene where 4 fragments located in regions of high nucleotide divergence appear to be low inducers of IFN-α/β response. Because the design of primers for the synthesis of RNA fragments was arbitrary (approximately every 500 bp), outliers may result from the elimination of either a secondary structure or a linear motif necessary for recognition in the host cell. Altogether, these results demonstrate that the most biased HIV RNA sequences are responsible for IFN-α/β stimulation.

### Subtype D is the Most Divergent Among HIV-1 Group M Viruses

**Figure 5:**
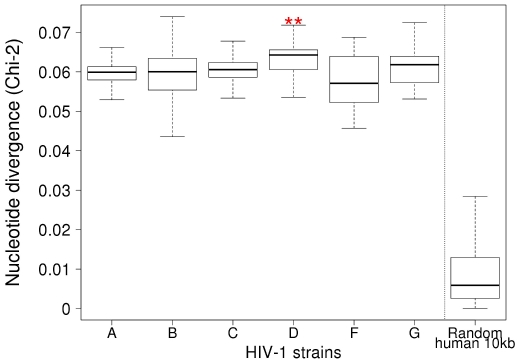
HIV-1 subtype D has the most divergent sequence. Boxplots show the median and dispersion of the nucleotide divergence of individual HIV-1 genomes from different subtypes to the average of human coding sequences. Red stars indicate that D subtype is significantly more divergent than other subtypes (p = 2×10^−6^ ANOVA test, p = 5.8×10^−6^ Student t-test, p = 2.8×10^−7^ Wilcoxon test). The random 10 kb column shows the median and standard deviation of the divergence between 10 kb of random human coding sequences and the average of all human coding sequences; 95% of the points have a divergence below 0.03, and less than 1% of the points are above 0.05, ie comparable to HIV divergence. Standard deviation gives a natural scale of variation to the graph. These data were not used in the statistical procedure to compare subtypes.

To investigate the possible influence of the biased nucleotide composition of HIV RNA in AIDS pathogenesis, we looked for an association between nucleotide composition of HIV-1 group M subtypes and their pathogenicity. Different studies recently reported that among HIV-1 group M subtypes, infection with subtype D is associated with a significantly faster CD4 cell decline than other subtypes [25], [26], [27], [28], thus indicating an increased pathogenicity of subtype D viruses. Many reasons may account for this increased pathogenicity, such as virus replicative capacity or escape to immune responses. To look for an association with nucleotide bias, we measured the divergence of nucleotide composition between all HIV-1 subtypes and human genome by computing the Chi-square distance between the A/C/G/U frequencies of more than 1300 HIV-1 complete genomes and the entire coding sequences of human genome (Figure 5). All HIV-1 group M “pure” subtypes, for which more than 20 genomic sequences are available in Los Alamos database (clade A, B, C, D, F & G), were used in this analysis. We first evaluated the divergence between all subtypes by using ANOVA test. Looking blindly among the subtypes highlights that at least one subtype is significantly more biased and more divergent relative to human genome than the others (p = 2.10^−6^). We then used the Student t-test to compare subtype D divergence relatively to all other subtypes pooled together. It appears that subtype D is significantly more divergent to human genes than all other subtypes (Bonferroni-corrected Student t-test, p = 3.10^−5^). The observation that the most pathogenic HIV-1 subtype is also the most divergent in nucleotide composition with human genome is a preliminary indication of a possible contribution of HIV-specific nucleotide bias in AIDS pathogenesis. Detecting this effect through large-scale comparative genomic analysis indicates that the causative mechanism involved is disseminated along the HIV-1 sequence.

## Discussion

Very little is known about the consequences of the biased nucleotide composition of human immunodeficiency viruses on their pathogenicity. In this report we addressed this question by analyzing the role of nucleotide composition of HIV-1-derived nucleic acids in stimulating IFN-α/β response, then by correlating the pathogenicity of HIV-1 M subtypes with the divergence of their nucleotide composition with human coding sequences.

To measure type-I IFN stimulation, we used an in vitro assay allowing to discriminating the role of RNA nucleotide composition in the absence of viral proteins. Our experiments show that the biased nucleotide composition of HIV RNA is detected in human cells and triggers a strong innate immune response. We evidenced that HIV-1-derived RNA molecules that are the most biased in their nucleotide composition as compared to human coding sequences are the most potent IFN-α/β stimulators. They correspond to the three main viral genes, which are the most abundant viral RNA molecules in the cytoplasm of infected cells during HIV viral production. Optimization of these sequences to human codon bias, and therefore to human A/C/G/U frequency abolished their stimulating capacity. Similarly, discrete fragments of 500 bp within HIV-1 genome stimulate type-I IFN according to their nucleotide composition, the most biased being the best stimulators. These results indicate the existence of a molecular mechanism capable of sensing the biased nucleotide composition of RNA molecules or its structural consequence, or the presence of A-rich linear motifs, such as instability elements for example. Several receptors may discriminate nucleic acids according to their nucleotide composition. HIV encodes multiple U-rich sequences recognized by Toll-like receptor 7/8 [29]. Cytosolic AT-rich DNA can be transcribed by RNA pol-III into RNA molecules that induce IFN-α/β through the RIG-I pathway [30]. In the HCV genome, RIG-I preferentially binds poly-U/UC, poly-U/UG and poly-A RNAs [31]. RIG-I is also able to trigger IFN-α/β response in the presence of genomic RNA extracted from HIV-1 particles [32]. Other hypotheses can be formulated, such as the formation of RNA secondary structures specifically recognized by innate immunity sensors, or the recognition of specific AU-rich linear motifs. Finally, sequence specific RNAse such as RNAse L could also increase the innate immune response against AU-rich RNAs [33].

Whether this nucleotide bias is detected in infected natural target cells and contributes to activating immune system in HIV patients is unknown. Therefore, we looked for an association between nucleotide composition of HIV-1 subtypes and their pathogenicity. To investigate whether HIV-1 subtype nucleotide composition, and particularly its divergence with the human genome, is associated with differential pathogenicity, we combined information from numerous HIV-1 complete genomes and from large-scale epidemiological studies [25], [26], [27], [28]. We observed that subtype D, which cause a faster CD4 cell decline, is significantly more divergent from the human genome than other subtypes. This observation supports the assumption that the higher capacity of biased HIV-1 nucleic acids to stimulate IFN-α/β synthesis may be linked with immune activation and pathogenesis. Although several other factors may determine the pathogenicity of a given HIV-1 subtype, such as replicative capacity, ability to escape adaptive immune response, restriction factors [12], [34], our data suggest that the strength of host innate immune response and its adaptation to viral nucleotide composition is critical.

To further extend this observation and determine whether the aggressive nucleotide composition of other lentiviruses than HIV-1 is linked to pathogenicity, it is of interest to look at non-human primate models for which SIV chronic viral infection is associated with either pathogenic or non-pathogenic outcome. Indeed, persistent infection by a highly replicating lentivirus does not necessarily lead to AIDS, and natural hosts such as African green monkeys (AGM), sooty mangabeys (SM) and mandrills (Mnd), never develop chronic immune activation and do not progress to AIDS, despite chronic high viremia [35] [4]. Conversely, experimental transmission of the SM type of SIV (SIVsm) to *rhesus* macaques (RM), or of SIVagm.ver90 and SIVagm.sab92018 to pigtailed macaques causes immune over-activation and progression to AIDS [36]. It may thus be hypothesized that interspecies transmission of SIV results in immune hyper-activation within new hosts, whereas natural hosts, in which the infection has long been established, tolerate the virus and control the homeostasis of their immune system. Analyzing the nucleotide divergence of different SIV strains with their primate hosts would be useful in this context. However, although numerous SIV complete genomes are available, only three primates complete genomes have been published (human, chimpanzee and rhesus macaque) and large-scale genomic sequences from natural SIV hosts primates, in which infection is non-pathogenic, are quite rare while they would provide a crucial input. We are currently working on such analyses.

In conclusion, our observations suggest for the first time that the specific nucleotide composition of HIV-1 genome might have a consequence on its pathogenicity. They suggest the existence of cellular immune mechanisms able to discriminate RNA sequences according to their nucleotide composition or to detect specific secondary structures or linear motifs within biased RNA sequences. These observations are valid at intra-genome and between-genome scales, indicating that the causative mechanism they involve is disseminated along the HIV-1 sequence, and therefore can only be detected through large-scale comparative genomic studies. Our observations also lead to readdress the antiviral role of RNA editing enzymes such as cytidine deaminases [37]. In the case of A3G, increasing the A/U content of lentiviral genomes makes the viral RNAs more divergent. The resulting sequences, if still able to replicate, would activate the innate immune response to a higher extent. During co-evolution between a lentivirus and its host, this mechanism might help the immune system to reinforce the discrimination between self and non-self to preserve the organism, counterbalancing the humanization of viral genome due to nucleotidic pool constraints in the host cell [19], [20]. Intriguingly, this mechanism might also contribute to maintaining or increasing the virus pathogenicity. Although further work is necessary to confirm this observation in non-human primate models of SIV infection, highlighting the role of lentiviral nucleotide composition may help in understanding lentiviral pathogenesis and in AIDS vaccine design.

## Materials and Methods

### RNA Preparation

Primers (Table S1) were designed to amplify 38 overlapping fragments of around 500 pb long by PCR reaction (Enzyme Phusion, 35 cycles, Ta = 60°C, 30 sec. elongation). Primers were also designed to amplify sequences (1 min. elongation) from Gag, Pol or Env from HIV-1 clone *hxb2* or from plasmid expressing codon-optimized sequences of the genes. Gag optimized gene was purchased from Genecust, Pol and Env genes were purchased to Geneart. A 5′ tail containing the sequence of T7 promoter was included in every forward primer to allow the subsequent *in vitro* transcription reaction. PCR products were purified and used as template for T7 RNA synthesis according to manufacturer’s instructions (T7 RiboMA Express, Promega). Resulting RNA were purified using RNeasy mini kit (Qiagen) and concentration was determined by nanodrop measurement. RNA quality and integrity was controlled using Agilent-2100 Bioanalyzer prior to cell transfection.

### Luciferase Reporter Gene Assay

Expression of IFN-α/β was determined by transient transfection of reporter plasmids pISRE-Luc containing five ISRE enhancer elements upstream of the firefly luciferase gene (Stratagene). HEK 293 T cells (ATCC, CRL11268) were plated in 24-well plates (2×10^5^ per well). One day later, cells were transfected using 1 µl of Lipofectamine 2000 (Invitrogen) with pISRE-Luc reporter plasmid (250 ng/well), a plasmid harboring a thymidine kinase (Tk) promoter just upstream of the renilla luciferase gene (25 ng/well), and 12 ng of each RNA fragment (500 pb) or 20 ng of Gag, Pol or Env (*hxb2* or optimized). After 20 h, cells were lysed, and the firefly and renilla luciferase activities were measured in cell lysates using the Dual-luciferase Reporter Assay System (Promega) according to manufacturer’s instructions. Reporter activity was calculated as a triplicate of the ratio of firefly luciferase activity to reference renilla luciferase activity.

### Data and Statistical Analyses

Chi-square distances between the nucleotide frequencies of HIV-1 and the human genome were computed by:



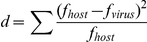



The estimate of the nucleotidic composition of the human genome was computed on all coding sequences. For HIV-1, the entire genome of the *hxb2* clone (HIVHXB2CG) was used for the frequencies estimation. All HIV-1 sequences from different subtypes (more than 1300) were downloaded from the HIV database (www.hiv.lanl.gov). Statistical procedure in the analysis of these data involved first an ANOVA to detect a difference between subtypes, followed by a Student t-test to compare subtype D relatively to all others subtypes pooled together. Bonferroni multiple test correction was applied. The correlation between Luciferase reporter activity and host/virus nucleotide divergence was computed using the nucleotide frequencies of each 500 bp RNA fragment using standard linear correlation test. Density of human genes of given length and nucleotidic divergence relative to the average of all human coding sequences (Fig. 1) was computed using a standard 2-dimensional gaussian kernel. All analyses were done using the R software.

## Supporting Information

Table S1 **HIV-1 primers used in** **Figures 3** **and** **4** **.**
(DOC)

Figure S1 **Efficiency of HEK 293T cells transfection in presence of HIV-1 RNA fragments.** RNA was co-transfected with a reporter ISRE-firefly luciferase plasmid and a plasmid harboring a thymidine kinase promoter upstream the renilla luciferase gene in triplicate experiments. As a control of transfection efficiency, the renilla luciferase activity was measured in cell lysates 20 hours after transfection. The different RNA fragments used are indicated.
(TIF)
