# Supplementary material for: The Biased Nucleotide Composition of HIV-1 Triggers Type I Interferon Response and Correlates with Subtype D Increased Pathogenicity
Source: PLoS One. 2012 Apr 18;7(4):e33502. doi: 10.1371/journal.pone.0033502 (PMC3329495; doi:10.1371/journal.pone.0033502)
Supplement: Table S1 — HIV-1 primers used in Figures 3 and 4 . (DOC) [file pone.0033502.s001.doc]

**Table S1. HIV-1 primers used in Figures 3-4**

| **Fragment** | **Forward Primer with T7 promoter** | **Reverse Primer** | **Position in *hxb2* genome** |
| --- | --- | --- | --- |
| 1 | TGTAATACGACTCACTATAGGGTGGAAGGGCTAATTCACTCCCAACG | GCAGTGGGTTCCCTAGTTAGC | 1-517 |
| 2 | TGTAATACGACTCACTATAGGGGAGAAGTGTTAGAGTGGAGGTTTGAC | CTCTCTCCTTCTAGCCTCCGCTA | 244-789 |
| 3 | TGTAATACGACTCACTATAGGGGCTAACTAGGGAACCCACTGC | CTGTCTGAAGGGATGGTTGTAGC | 497-1000 |
| 4 | TGTAATACGACTCACTATAGGGTAGCGGAGGCTAGAAGGAGAGAG | CTGGGCTGAAAGCCTTCTCTT | 767-1288 |
| 5 | TGTAATACGACTCACTATAGGGGCTACAACCATCCCTTCAGACAG | CATCCATCCTATTTGTTCCTGAAGGG | 978-1539 |
| 6 | TGTAATACGACTCACTATAGGGAAGAGAAGGCTTTCAGCCCAG | GGACCAACAAGGTTTCTGTCATCCAA | 1268-1759 |
| 7 | TGTAATACGACTCACTATAGGGCCCTTCAGGAACAAATAGGATGGATG | CTGCAATTTCTGGCTGTGTGC | 1514-2006 |
| 8 | TGTAATACGACTCACTATAGGGTTGGATGACAGAAACCTTGTTGGTCC | CCAAAGAGTGACCTGAGGGAAG | 1734-2270 |
| 9 | TGTAATACGACTCACTATAGGGGCACACAGCCAGAAATTGCAG | ATGTTGACAGGTGTAGGTCCTACTA | 1986-2503 |
| 10 | TGTAATACGACTCACTATAGGGCTTCCCTCAGGTCACTCTTTGG | GGCAAATACTGGAGTATTGTATGGATT | 2249-2735 |
| 11 | TGTAATACGACTCACTATAGGGTAGTAGGACCTACACCTGTCAACAT | GGTGATCCTTTCCATCCCTGTGGA | 2479-3019 |
| 12 | TGTAATACGACTCACTATAGGGAATCCATACAATACTCCAGTATTTGCC | GAGTTCATAACCCATCCAAAGGAATG | 2709-3251 |
| 13 | TGTAATACGACTCACTATAGGGTCCACAGGGATGGAAAGGATCACC | TGATGGGTCATAATACACTCCATGTAC | 2996-3515 |
| 14 | TGTAATACGACTCACTATAGGGCATTCCTTTGGATGGGTTATGAACTC | CTTGCCAATACTCTGTCCACCATG | 3226-3771 |
| 15 | TGTAATACGACTCACTATAGGGGTACATGGAGTGTATTATGACCCATCA | GTTTACTTCTAATCCCGAATCCTGCAA | 3489-4031 |
| 16 | TGTAATACGACTCACTATAGGGCATGGTGGACAGAGTATTGGCAAG | CATCTTGGGCCTTATCTATTCCATCT | 3748-4260 |
| 17 | TGTAATACGACTCACTATAGGGTTGCAGGATTCGGGATTAGAAGTAAAC | CCTGCCCTGTTTCTGCTGGAAT | 4005-4515 |
| 18 | TGTAATACGACTCACTATAGGGAGATGGAATAGATAAGGCCCAAGATG | GTACTGCTGTCTTAAGATGTTCAGCC | 4235-4758 |
| 19 | TGTAATACGACTCACTATAGGGATTCCAGCAGAAACAGGGCAGG | CTTGGCACTACTTTTATGTCACTATTATC | 4494-5014 |
| 20 | TGTAATACGACTCACTATAGGGGGCTGAACATCTTAAGACAGCAGTAC | CCAGTCTCTTTCTCCTGTATGCAG | 4733-5277 |
| 21 | TGTAATACGACTCACTATAGGGGATAATAGTGACATAAAAGTAGTGCCAAG | GCTGCTAGTGCCAAGTATTGTAGAG | 4986-5495 |
| 22 | TGTAATACGACTCACTATAGGGCTGCATACAGGAGAAAGAGACTGG | CTTATTATGGCTTCCACTCCTGCC | 5254-5743 |
| 23 | TGTAATACGACTCACTATAGGGCTCTACAATACTTGGCACTAGCAGC | GCTCTTCGTCGCTGTCTCC | 5471-6003 |
| 24 | TGTAATACGACTCACTATAGGGGGCAGGAGTGGAAGCCATAATAAG | CTCCACAAGTGCTGATATTTCTCCT | 5720-6259 |
| 25 | TGTAATACGACTCACTATAGGGGGAGACAGCGACGAAGAGC | TTCTGTCACATTTACCAATACTACTTCTT | 5985-6497 |
| 26 | TGTAATACGACTCACTATAGGGAGGAGAAATATCAGCACTTGTGGAG | CTTTCTGCACCTTACCTCTTATGCT | 6235-6738 |
| 27 | TGTAATACGACTCACTATAGGGAAGAAGTAGTATTGGTAAATGTGACAGAA | CCTCTTCTTCTGCTAGACTGCCATTTA | 6469-7032 |
| 28 | TGTAATACGACTCACTATAGGGAGCATAAGAGGTAAGGTGCAGAAAG | CAAATTGTTCTCTTAATTTGCTAGCTATCTG | 6714-7284 |
| 29 | TGTAATACGACTCACTATAGGGTAAATGGCAGTCTAGCAGAAGAAGAGG | CTGCATGGGAGGGTGATTGT | 7006-7480 |
| 30 | TGTAATACGACTCACTATAGGGCAGATAGCTAGCAAATTAAGAGAACAATTTG | CTCTCTGCACCACTCTTCTCTTT | 7254-7749 |
| 31 | TGTAATACGACTCACTATAGGGACAATCACCCTCCCATGCAG | GGTATCTTTCCACAGCCAGGA | 7461-7983 |
| 32 | TGTAATACGACTCACTATAGGGAAAGAGAAGAGTGGTGCAGAGAG | CCAATTCCACAAACTTGCCCA | 7727-8240 |
| 33 | TGTAATACGACTCACTATAGGGTCCTGGCTGTGGAAAGATACC | CAGATCGTCCCAGATAAGTGCC | 7963-8504 |
| 34 | TGTAATACGACTCACTATAGGGTGGGCAAGTTTGTGGAATTGG | CAAGCCCTGTCTTATTCTTCTAGGTA | 8220-8777 |
| 35 | TGTAATACGACTCACTATAGGGGGCACTTATCTGGGACGATCTG | CCACCTCCTCCTCCTCTTGT | 8483-8995 |
| 36 | TGTAATACGACTCACTATAGGGTACCTAGAAGAATAAGACAGGGCTTG | CTCTCCTTTATTGGCCTCTTCTATCTT | 8752-9276 |
| 37 | TGTAATACGACTCACTATAGGGACAAGAGGAGGAGGAGGTGG | GGATCTGAGGGCTCGCCACT | 8976-9507 |
| 38 | TGTAATACGACTCACTATAGGGAAGATAGAAGAGGCCAATAAAGGAGAG | TGCTAGAGATTTTCCACACTGACT | 9250-9719 |
| Gag | TGTAATACGACTCACTATAGGGTGGGTGCGAGAGCGTCAGTATTAAGC | TTACTGGCTGCTGGGGTCGTTGC | 791-2294 |
| Pol | TGTAATACGACTCACTATAGGGTGGATGGCCCAAAAGTTAAACAATGGCC | TCATGTCCATTTATCAGGATGGAGTTCATAACC | 2598-3269 |
| Env | TGTAATACGACTCACTATAGGGTGAGAGTGAAGGAGATCAGGAGGAATTATC | TTATAGCAAAAGCCTTTCCAAGCCCTGTC | NS |
| Gag opt | TGTAATACGACTCACTATAGGGTGGCCGCCAGAGCCAGCATC | TCAGTTGGCCTGCCTCTCGGTG | NS |
| Pol opt | TGTAATACGACTCACTATAGGGTGGACGGCCCTAAGGGTGAAACAG | TCAGGTCCACTTGTCGGGGTGCAG | NS |
| Env opt | TGTAATACGACTCACTATAGGGTGCGCGTGAAGGAGATCCGGC | TCACAGCAGCAGCCTCTCCAGG | NS |
