# Supplementary figures and images for: The Biased Nucleotide Composition of HIV-1 Triggers Type I Interferon Response and Correlates with Subtype D Increased Pathogenicity
Source: PLoS One. 2012 Apr 18;7(4):e33502. doi: 10.1371/journal.pone.0033502 (PMC3329495; doi:10.1371/journal.pone.0033502)

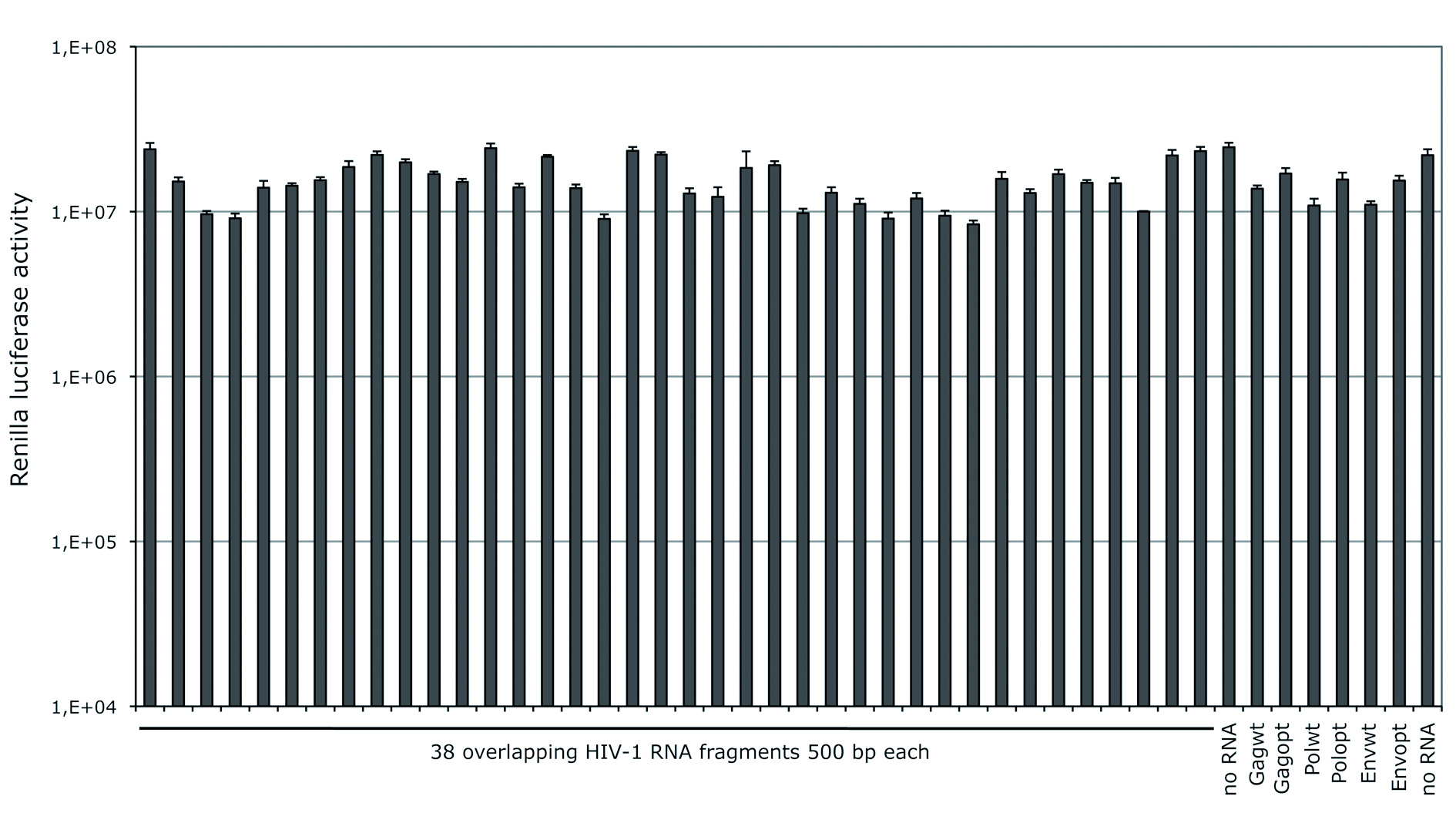

Supplement: Figure S1 — Efficiency of HEK 293T cells transfection in presence of HIV-1 RNA fragments. RNA was co-transfected with a reporter ISRE-firefly luciferase plasmid and a plasmid harboring a thymidine kinase promoter upstream the renilla luciferase gene in triplicate experiments. As a control of transfection efficiency, the renilla luciferase activity was measured in cell lysates 20 hours after transfection. The different RNA fragments used are indicated. (TIF) [file pone.0033502.s002.tif]
